# Association Between Patient Portal Use and Perceived Patient-Centered Communication Among Adults With Cancer: Cross-sectional Survey Study

**DOI:** 10.2196/34745

**Published:** 2022-08-09

**Authors:** Maryum Zaidi, Daniel J Amante, Ekaterina Anderson, Mayuko Ito Fukunaga, Jamie M Faro, Christine Frisard, Rajani S Sadasivam, Stephenie C Lemon

**Affiliations:** 1 Division of Preventive and Behavioral Medicine Department of Population and Quantitative Health Sciences UMass Chan Medical School Worcester, MA United States; 2 Division of Health Informatics and Implementation Science Department of Population and Quantitative Health Sciences UMass Chan Medical School Worcester, MA United States; 3 Center for Healthcare Organization and Implementation Research Veterans Affairs Bedford Healthcare System Bedford, MA United States; 4 Department of Medicine UMass Chan Medical School Worcester, MA United States

**Keywords:** health information technology, informatics, cancer care, patient-centered communication, patient portal, patient communication, cancer, oncology, health information, information seeking, patient-centered care, patient perception

## Abstract

**Background:**

Patient-centered communication (PCC) plays a vital role in effective cancer management and care. Patient portals are increasingly available to patients and hold potential as a valuable tool to facilitate PCC. However, whether more frequent use of patient portals is associated with increased perceived PCC and which mechanisms might mediate this relationship have not been fully studied.

**Objective:**

The goal of this study was to investigate the association between the frequency of access of patient portals and perceived PCC in patients diagnosed with cancer. We further sought to examine whether this association was mediated by patients’ self-efficacy in health information–seeking.

**Methods:**

We used data from the Health Information National Trend Survey 5 (HINTS 5) cycle 3 (2019) and cycle 4 (2020). This analysis includes 1222 individuals who self-reported having a current or past diagnosis of cancer. Perceived PCC was measured with a 7-item HINTS 5–derived scale and classified as low, medium, or high. Patient portal use was measured by a single item assessing the frequency of use. Self-efficacy about health information–seeking was assessed with a 1-item measure assessing confidence in obtaining health information. We used adjusted multinomial logistic regression models to estimate relative risk ratios (RRRs)/effect sizes of the association between patient portal use and perceived PCC. Mediation by health information self-efficacy was investigated using the Baron and Kenny and Karlson-Holm-Breen methods.

**Results:**

A total of 54.5% of the sample reported that they had not accessed their patient portals in the past 12 months, 12.6% accessed it 1 to 2 times, 24.8% accessed it 3 to 9 times, and 8.2% accessed it 10 or more times. Overall, the frequency of accessing the patient portal was marginally associated (*P*=.06) with perceived PCC in an adjusted multinominal logistic regression model. Patients who accessed their patient portal 10 or more times in the previous 12 months were almost 4 times more likely (RRR 3.8, 95% CI 1.6-9.0) to report high perceived PCC. In mediation analysis, the association between patient portal use and perceived PCC was attenuated adjusting for health information–seeking self-efficacy, but those with the most frequent patient portal use (10 or more times in the previous 12 months) were still almost 2.5 times more likely to report high perceived PCC (RRR 2.4, 95% CI 1.1-5.6) compared to those with no portal use.

**Conclusions:**

Increased frequency of patient portal use was associated with higher PCC, and an individual’s health information–seeking self-efficacy partially mediated this association. These findings emphasize the importance of encouraging patients and providers to use patient portals to assist in patient-centeredness of cancer care. Interventions to promote the adoption and use of patient portals could incorporate strategies to improve health information self-efficacy.

## Introduction

Approximately 17 million people in the United States are living with cancer [1]. Cancer, in many forms, is considered a chronic disease [2]. Living with cancer imposes significant disease management demands and carries substantial psychological, financial, and physical burdens [3]. Patients undergoing active treatment and survivors needing continued cancer surveillance and management deserve high-quality patient-centered care rooted in respect for patients’ dignity and clear communication [4,5]. There has been a strong and growing emphasis in policy and practice on patient-centered care since the Institute of Medicine released a consensus report in 2013 that provided a blueprint for it [6].

Patient-centered care comprises multiple factors, and patient-centered communication (PCC) is an essential aspect [7,8]. PCC is a communication style that seeks to understand and account for the patient’s concerns, needs, feelings, and psychosocial and cultural context [9,10]. PCC increases patient satisfaction in chronic disease management, especially in cancer care [5,9-13]. However, PCC is challenging and time-consuming in practice [14] and can benefit from patient-facing digital health tools that can aid in effective communication within the time restraints of busy oncology settings [11]. Patient portals are potentially one such tool. They enable patients to view their medical records, communicate via secure messaging with their care teams, access lab results, renew prescriptions, request appointments, and pay their medical bills [15,16]. Even though patient portals have been documented to improve patient engagement, increase PCC, advance health care quality, and improve psychosocial outcomes in medical care [17-19], their optimal use in cancer care delivery has not yet been achieved [20-22].

Much research promoting PCC in cancer care has focused on assessing and improving clinicians’ skills and training. Less work, however, has been done on patient-specific characteristics such as a patient’s ability to seek information [23]. One specific factor impacting the quality of care received in cancer care is the patient’s perceived self-efficacy [24]. Perceived self-efficacy is one’s confidence to exercise control over one’s functioning and execute actions that will lead to a specific outcome [25]. It influences the adoption and maintenance of health-promoting behaviors [23,26]. Self-efficacy related to one’s ability to take care of one’s health has shown a positive association in earlier studies with PCC [27], including in a study of patients diagnosed with cancer [28]. Moreover, self-efficacy has been shown to mediate the association between PCC and emotional distress in patients diagnosed with cancer [29].

Health information self-efficacy is a personal belief that one can take action to get the information if they need it regarding a health concern [30]. Patients diagnosed with cancer have an increased need for information-seeking due to the level of health care decisions they need to make [31]. Providers remain the most trusted form of knowledge in cancer information-seeking [32-34]. Health informatics tools such as patient portals have become additional channels by which patients communicate with their providers and access their medical records [35,36]. Patients with increased health information self-efficacy may be better positioned to engage with their clinical team through patient portals, potentially leading to better rapport and better perceived patient-centeredness of communication. However, this has yet to be empirically studied.

The purpose of this study was to assess the association between the frequency of access to patient portals and perceived PCC in a national sample of individuals who have had a diagnosis of cancer. We further sought to determine whether self-efficacy related to information-seeking mediated the relationship between frequency of access to patient portals and PCC. We hypothesized that greater frequency of portal access would be associated with high PCC and that health information self-efficacy mediates the relationship between portal use frequency and PCC.

## Methods

### Data Source

Data examined for this study were from the Health Information National Trends Survey (HINTS). HINTS is a cross-sectional survey that the National Cancer Institute has regularly administered since 2004. HINTS aims to assess how people access and use health information, how people use information technology to manage health and health information, and the degree to which people are engaged in healthy behaviors [37]. The population from which HINTS samples is civilian, noninstitutionalized adults aged 18 years and above living in the United States. Similar to prior HINTS cycles, the sampling frame consisted of drawing on a database of participant addresses used by the Marketing System Group to provide random samples of addresses [38].

This study combines the third and fourth data collection cycles for HINTS 5. HINTS 5 cycle 3 was conducted from January 22 to April 30, 2019, and it consisted of data from 3500 respondents using a mailed survey. The response rate for the mailed survey was 30.2%. During HINTS 5 cycle 3, a web pilot test was run alongside the self-administered mailed version from January 29 to May 7, 2019. The web pilot comprised 2046 additional respondents. The web-based pilot included an experiment testing the effectiveness of offering a $10 Amazon gift card for responding via the web. Web pilot respondents who were offered the bonus incentive had a slightly higher response rate (31.5%) compared to the control group (29.6%), who did not receive the Amazon gift card [38]. We used both mail-in and online responses for HINTS 5 cycle 3. To use the combined sample, we tested for the differences in both versions for our outcome variable by mode and found no difference. The data collection for HINTS 5 cycle 4 was conducted from February 24, 2020, to June 15, 2020, using self-administered mail-in surveys only. A total of 3865 surveys were collected. The overall response rate for HINTS 5 cycle 4 was 32.6% [39]. Of the 9411 HINTS 5 participants in cycles 3 and 4, 1482 self-reported a diagnosis of cancer, the population of interest for this study. Of these individuals, 260 were excluded due to missing data, resulting in a final analytic sample of 1222.

### Ethics Approval

This study qualified for exempt status from the Committee for the Protection of Human Subjects at the University of Massachusetts Chan Medical School.

### Measures

#### Use of Patient Portals

Use of patient portals was measured by the question: How many times did you access your online medical record in the last 12 months? We categorized this as no use, 1 to 2 times, 3 to 9 times, and 10 or more times during the last 12 months. Online medical records are accessed with the help of patient portal secure log-ins [40-42], and patient portal is a more familiar term [16]; hence we used the term patient portal in this paper for this measure.

#### Perceived PCC

Perceived PCC was assessed with 7 items. Participants asking about communication with all health professionals were asked to assess the frequency with which their providers engaged in the following behaviors in the past 12 months: Give you the chance to ask all the health-related questions you had? Give the attention you need to your feelings and emotions? Involve you in decisions about your health care as much as you wanted? Make sure you understood the things you needed to do to take care of your health? Explain things in a way you could understand? Spend enough time with you? Help you deal with feelings of uncertainty about your health or health care? All items were measured on a 4-point Likert scale ranging from always (1) to never (4).

To create the PCC score, items were reverse coded so that higher numbers reflected higher levels of communication. The mean of all 7 items is transformed to a linear scale ranging from 1 to 100 [11]. The PCC score for individuals in our study was highly skewed with a great number of individuals at the top of the scale toward higher communication. As such, we broke the scale into 3 categories: low PCC (<25th percentile, mean 51.7, SE 2.0, range 0-66.7); moderate PCC (25th-50th percentile, mean 78.2, SE 0.7, range 71.4-85.7), and high PCC (≥50th percentile, mean 97.9, SE .3, range 86.7-100).

#### Health Information–Seeking Self-efficacy

The mediating variable was health information–seeking self-efficacy. We hypothesized that it mediated the relationship between frequency of portal use and perceived PCC. Self-efficacy in seeking health information was measured using 1 item used in previous studies [43,44]. In cycle 3, this item was worded as such: Overall, how confident are you that you could get advice or information about health or medical topics if you needed it? This question was worded differently in cycle 4: Overall, how confident are you that you could get advice or information about cancer if you needed it? In both cycles, the answer choices used a Likert scale ranging from 1 to 5, from completely confident (1) to not confident at all (5). We treated them as the same question in our analyses as our sample consisted of only patients with a diagnosis of cancer. Because of small cell sizes, response choices were dichotomized to somewhat/a little/not at all confident versus completely/very confident and conceptualized as highly confident versus not highly confident. This dichotomization is similar to that used in a previous study using this variable [45].

#### Other Variables

Our analysis is adjusted for gender (male, female), age (<55 years, 55 to 64 years, 65 to 74 years, 75 years and older), race/ethnicity (non-Hispanic White, non-Hispanic Black, Hispanic, non-Hispanic Asian/other), income level (<$35,000, $35,000-$99,999, ≥$100,000), education level (less than high school, high school graduate, some college, college graduate or more), and health insurance status (private, Medicare, Medicaid, or dual coverage). Previous research has shown that these variables have an impact on access and use of patient portals [20,46,47]. In this analysis, we also accounted for time since diagnosis of cancer (less than 1 year, 2 to 5 years, 6 to 10 years, ≥11 years) as it can also impact a patient’s information-seeking needs [48].

### Statistical Analysis

All analyses used Taylor series variance estimation with HINTS sampling weights to produce nationally representative estimates as suggested in HINTS methodology guides [38,39]. Characteristics of the sample were described using weighted percentages. Multivariable multinomial logistic regression models estimated relative risk ratios (RRRs)/effect sizes and 95% confidence intervals comparing high and moderate perceived PCC versus low perceived PCC. We tested 2 regression models, one without health information self-efficacy and one with it. Models were adjusted for gender, age, race/ethnicity, income, education, type of health insurance, time since diagnosis, and HINTS cycle. The role of self-efficacy as a mediator of the association between frequency of access to patient portals with PCC was first investigated using the Baron and Kenny method [49,50]. A formal mediation analysis using the Karlson-Holm-Breen method was then conducted to estimate and interpret total direct and indirect effects for nonlinear probability modes [51]. All analyses were conducted using Stata 14 (StataCorp LLC).

## Results

### Sample Description

The analytic sample with complete data responses included 1222 respondents, 661 from HINTS 5 cycle 3 and 561 from HINTS 5 cycle 4. As shown in Table 1, about half (49.1%) of the sample was younger than 65 years and male (45.4%). A majority (77.0%) were non-Hispanic White, 41.2% reported less than $35,000 in household income, and approximately 70% attended college. Consistent with our categorization scheme, approximately one-quarter of respondents were categorized as low (26.5%) or moderate (24.5%) on the PCC scale and slightly less than half (49.0%) were categorized as high. About half (54.49%) had not accessed their patient portal in the past 12 months. In this sample, the greatest proportion of those with no portal use were females (55.5%), aged 75 years and older (71.7%), non-Hispanic Black ( 77.1%), <$35,000 per year in income (64.9%), with less than high school education (76.5%). Almost two-thirds (62.6%) of the sample reported high levels of health information–seeking self-efficacy.

**Table 1:** Characteristics and differences in portal use among respondents with a self-reported cancer diagnosis in the Health Information National Trends Survey cycles 3 and 4 (n=1222 weighted percentages)^a^.

Characteristic		Total sample, %	Portal use in the past 12 months, %				
			No use	1-2 times	3-9 times	≥10 times	*P* value
**Gender**		—^b^	—	—	—	—	.31
	Male	45.4	50.9	17.2	23.5	8.4	—
	Female	54.6	55.5	9.8	26.3	8.3	—
**Age group (years)**		—	—	—	—	—	<.001
	<55	26.5	52.0	11.5	29.7	6.9	—
	55-64	22.6	41.1	15.6	25.6	17.8	—
	65-74	24.0	49.6	17.5	25.6	7.3	—
	≥75	26.9	71.7	5.1	20.2	2.9	—
**Race/ethnicity**		—	—	—	—	—	.02
	Non-Hispanic White	77.0	46.5	16.0	27.1	10.4	—
	Non-Hispanic Black	7.4	77.1	7.7	8.7	6.5	—
	Hispanic	10.8	66.2	6.8	25.7	1.4	—
	Non-Hispanic Asian/other	4.9	54.1	8.5	34.2	3.2	—
**Income level ($)**		—	—	—	—	—	.03
	<35,000	41.2	64.9	7.9	18.8	8.5	—
	35,000-99,999	39.7	49.9	15.1	27.5	7.5	—
	>100,00	19.1	41.6	17.5	32.0	9.0	—
**Highest level of education**		—	—	—	—	—	<.001
	Less than high school	7.9	76.5	1.2	21.9	0.4	—
	High school graduate	22.2	61.6	10.3	21.7	6.5	—
	Some college	42.6	58.6	10.5	21.5	9.4	—
	College graduate or higher	27.3	34.1	21.6	34.2	10.2	—
**Health insurance**		—	—	—	—	—	.17
	Private (employer or purchased on own)	56.3	50.1	15.8	25.5	8.7	—
	Medicare and privately purchased insurance	8.8	55.0	11.2	28.8	5.1	—
	Medicare	25.0	62.0	8.2	24.7	5.1	—
	Medicaid	7.9	54.0	8.8	15.7	21.5	—
	Other/IHS^c^/VA^d^/Tricare	2.0	82.3	4.3	13.4	0	—
**Time since diagnosis (year)**		—	—	—	—	—	.21
	<1	15.9	48.8	12.3	25.0	13.9	—
	2-5	21.2	47.9	11.7	24.8	15.5	—
	6-10	15.5	60.9	10.2	21.6	7.3	—
	≥11	47.4	53.5	13.5	28.8	4.2	—
**Patient-centered communication score**		—	—	—	—	—	.19
	Low (<25th percentile)	26.5	61.2	15.0	20.5	3.4	—
	Moderate (25th-50th percentile)	24.5	53.0	10.6	29.5	7.1	—
	High (≥50th percentile)	49.1	51.7	12.2	24.8	11.3	—
**Health information–seeking self-efficacy**		—	—	—	—	—	.006
	Somewhat/a little/not at all	37.4	63.0	13.0	20.7	3.3	—
	Completely/very	62.6	48.8	12.5	27.6	11.1	—

^a^All analyses used Taylor Series variance estimation with Health Information National Trends Survey sampling weights to produce nationally representative estimates.

^b^Not applicable.

^c^IHS: Indian Health Service.

^d^VA: Veterans Affairs.

### Multivariable Multinomial Model

Results of the multinomial model assessing the association between frequency of portal use and perceived PCC are presented in the middle column of Table 2. In the overall multivariable multinomial model, the frequency of access to the patient portal was marginally associated (*P*=.06) with PCC. Patients who accessed their patient portal only 1 or 2 times were equally as likely to have moderate PCC versus low PCC (RRR 0.99, 95% CI 0.42-2.34) than those who did not access it. Those who accessed the patient portal 3 to 9 times had more than twice the odds of moderate versus low PCC (RRR 2.22, 95% CI 1.01-4.86) than those who never accessed it. Those who accessed the patient portal 10 or more times were almost 3 times as likely to have moderate PCC versus low PCC (RRR 2.91, 95% CI 0.89-9.49) than those who did not access it. With respect to comparisons between respondents with high PCC versus low PCC, those who accessed the patient portal 1 or 2 times were 14% more likely than those who did not access it to have high versus low PCC (RRR 1.14, 95% CI 0.49-2.64). Those who accessed it 3 to 9 times had a 67% increase in the odds of high PCC versus low PCC (RRR 1.67, 95% CI 0.88-3.16). Last, those who accessed their record 10 or more times were almost 4 times more likely to have high PCC versus low (RRR 3.63, 95% CI 1.58-8.34).

**Table 2:** Results of adjusted multinomial logistic regression models measuring the association of frequency of online access to patient portals with perceived patient-centered communication score^a^.

Characteristic		Without adjustment for health information–seeking self-efficacy				With adjustment for health information–seeking self-efficacy		
		Moderate vs low PCC^b^, RRR^c^ (95% CI)	High vs low PCC, RRR (95% CI)	*P* value	Moderate vs low PCC, RRR (95% CI)		High vs low PCC, RRR (95% CI)	*P* value
**Frequency of patient portal access**		—^d^	—	.06	—		—	.25
	None	—	—	—	—		—	—
	1-2 times	0.99 (0.42-2.34)	1.14 (0.49-2.64)	—	0.94 (0.39-2.23)		0.94 (0.38-2.32)	—
	3-9 times	2.22 (1.01-4.86)	1.67 (0.88-3.16)	—	2.01 (0.91-4.48)		1.31 (0.67-2.56)	—
	≥10 times	2.91 (0.89-9.49)	3.63 (1.58-8.34)	—	2.49 (0.78-8.02)		2.32 (1.03-5.23)	—
**Health information–seeking self-efficacy**		—	—	—	—		—	<.001
	Somewhat/a little/not at all	—	—	—	1.78 (0.97-3.26)		4.57 (2.57-8.12)	—
	Completely/very high	—	—	—	1.78 (0.97-3.26)		4.57 (2.57-8.12)	—

^a^All analyses adjust for gender, age, race/ethnicity, income level, education level, health insurance status, and time since diagnosis.

^b^PCC: patient-centered communication.

^c^RRR: relative risk ratio.

^d^Not applicable.

### Mediation Analyses

The 4-step Baron and Kenny method was first used to investigate the role of health information–seeking self-efficacy as a mediator of the association between frequency of patient portal use and PCC [52]. In multinomial logistic models, we found the frequency of patient portal use overall was marginally associated with PCC (step 1, column 1 of Table 2, *P*=.06). Of note, those who accessed their portal 10 or more times (compared to those who did not access it) were more likely to have high PCC versus low PCC (RRR 3.63, 95% CI 1.58-8.34). We also found that the frequency of patient portal use was significantly associated with health information–seeking self-efficacy (step 2, column 2 of Table 2, *P*<.001). Health information–seeking self-efficacy was also associated with PCC. Individuals with high self-efficacy were more likely to report high PCC compared to those with low self-efficacy (step 3, column 3 of Table 3; RRR 4.57, 95% CI 1.03-5.23). When adjusting for health information self-efficacy, the *P* value for the association of frequency of portal use and PCC was no longer marginally significant (step 4, column 3 of Table 2, *P*=.25). The association between portal use of 10 or more times (compared to none) was also attenuated but remained statistically significant, with those reporting high use more likely to report high PCC (RRR 2.23, 95% CI 1.03-5.23).

These findings led to a more formal mediation analysis using the Karlson-Holm-Breen method, presented in Table 3. This analysis revealed that all levels of patient portal use showed a decreased association with PCC when controlled for health information–seeking self-efficacy. The full results are presented in Table 3. In the Karlson-Holm-Breen analysis, for those who accessed the patient portal 10 or more times, the odds of having high PCC versus low PCC were almost 4 times greater than those who did not access the portal (95% CI 1.63-9.59). After controlling for health information–seeking self-efficacy, that effect decreased to 2.3 times (95% CI 0.94-5.72). A total of 43% of the association between portal use and PCC was due to health information–seeking self-efficacy.

**Table 3:** Mediation results of communication scores using the Karlson-Holm-Breen method.

Characteristic				Odds ratio (95% CI)		Confounding ratio		Mediated proportion (indirect/total)
**Frequency of patient portal access: moderate compared with lowest scores**								
	None		—^a^		—		—	
	**1-2 times**		—		–0.25		1.07	
		Total effect	1.02 (0.43-2.43)		—		—	
		Direct effect	0.94 (0.39-2.23)		—		—	
		Indirect effect	1.09 (0.92-1.28)		—		—	
	**3-9 times**		—		1.15		0.5	
		Total effect	2.23 (1.08-4.63)		—		—	
		Direct effect	2.01 (0.97-4.16)		—		—	
		Indirect effect	1.11 (0.93-1.33)		—		—	
	≥**10 times**		—		1.22		0.4	
		Total effect	3.05 (1.02-9.10)		—		—	
		Direct effect	2.49 (0.82-7.55)		—		—	
		Indirect effect	1.22 (0.95-1.58)		—		—	
**Frequency of patient portal access: highest compared with lowest scores**								
	None		—		—		—	
	**1-2 times**		—		–2.63		1.06	
		Total effect	1.17 (0.49-2.81)		—		—	
		Direct effect	0.94 (0.39-2.28)		—		—	
		Indirect effect	1.24 (0.85-1.81)		—		—	
	**3-9 times**		—		2		0.76	
		Total effect	1.73 (0.89-3.33)		—		—	
		Direct effect	1.31 (0.68-2.54)		—		—	
		Indirect effect	1.31 (0.89-1.93)		—		—	
	≥**10 times**		—		1.63		0.43	
		Total effect	3.95 (1.63-9.59)		—		—	
		Direct effect	2.32 (0.94-5.72)		—		—	
		Indirect effect	1.70 (1.11-2.60)		—		—	

^a^Not applicable.

## Discussion

### Principal Findings

This study examined the association between the frequency of patient portal use and perceived PCC in patients diagnosed with cancer. We also investigated health information–seeking self-efficacy as a mediator of this association. Our findings indicated that the frequent levels of patient portal use (≥10 times in the past year) may be correlated with high levels of PCC. We also found that this association was partially mediated by health information–seeking self-efficacy.

In cancer care delivery, patient portal use has been increasing [20]. Patients report having more self-advocacy by feeling more involved and informed in their care when they access information through patient portals. The use of portals allowed them to reach their providers in a timely manner and enhanced their participation in their in-person consultations [21]. Our findings suggest that frequency of portal use may have an important role to play in improving PCC with their providers. These findings further support the small body of literature that has demonstrated that patient portals positively impact patient communication with their providers in cancer care delivery [21,53-55].

The provider’s role is critical in establishing PCC, and patient portals are intended to enhance, not replace, patient-provider face-to-face interactions [54,56]. Prior work has demonstrated that such use cannot always replace the human approach needed for establishing PCC for everyone [55,57,58]. The portal use would facilitate patient-provider communication between visits and may better prepare patients with information for in-person visits. As patient portals become more widely used in all medical settings, cancer care providers, particularly given the complexity of cancer and its treatment [54], will need to become more engaged with how patients view their medical information. It will be beneficial to consider the portal within the framework of patient-centered care by valuing patient communication preferences [21]. However, these efforts will require health systems to enable the providers to have the time and reimbursement ability to allow for safe and effective integration of patient portal–related tasks in their daily workflow [59].

Although there was a strong association between high use of patient portals and PCC in this study, only a small proportion of the included sample were frequent users of patient portals, and more than half of the sample reported no patient portal use. The greatest proportion of those with no portal use were females, participants in the 75 years and older age group, non-Hispanic Black participants, in households with <$35,000 per year in income, and participants who reported to have had less than a high school education (76.5%). Our findings are consistent with prior research on these sociodemographic differences except for gender, where males were reported to be less likely to use patient portals in previous studies [60,61]. A future study will be fruitful in addressing low access to patient portals in oncology-specific populations focused on patient preferences, type, and stage of their cancer, along with their patient portal accessibility and other sociodemographic characteristics.

Our analysis further confirms that a significant digital divide persists in actively getting patients to engage with patient portals, as previously reported [62,63]. Patient portal technology may create or exacerbate health equity concerns by not addressing the divide that social determinants of health play in its access and use [64,65]. One promising action to reduce such disparities in portal use is to aim for universal access to health information technology tools and to become aware of users’ health literacy levels and preferred ways of communicating with the providers [66]. While provider encouragement is one of the factors associated with increased access and use of patient portals [67-70], referrals vary by patient race, socioeconomic status, and providers’ personal beliefs about the benefits of patient portal use, contributing significantly to access disparities [22,71]. Targeting providers with additional patient portal referral training could be an effective strategy for increasing patient portal adoption among cancer patients, as demonstrated in studies of other patient populations [72-74].

This analysis also demonstrated that health information–seeking self-efficacy partially mediates the association between patient portal use and PCC. Hence, our findings suggest that enhancing self-efficacy in portal use is an important intervention target. It is increasingly emphasized to incorporate user perspectives in health information technology designs [75]. Numerous ventures have incorporated patient-centered approaches in patient portal use [72,73]. One approach to accomplishing this in cancer care is to design portals according to the needs of patients with different kinds of cancers, as portal enrollment by cancer sites varies [76]. Research shows that the digital divide is not caused only by a lack of devices and knowledge but also by a lack of fit between digital tools and people’s experiences [66]. Hence, there remains a need to improve portals to increase confidence in user usability, including among underresourced populations and in populations that experience poor self-reported health, where portal use is reported to be beneficial [77-79]. For example, features such as OpenNotes, which allow patients to access provider notes via portals, have shown promise in increasing feelings in patients of being informed and in control of their care, thus increasing trust in clinicians [57,77-80]. Oncology patients who face a greater information burden have shown enthusiasm for reading their clinicians’ notes [22]. Another approach to increase patient portal use in cancer care is promoting interventions targeting portal awareness and supporting patients accessing their notes.

It is crucial to consider that enhancing portal use is not only dependent on increasing competencies such as knowledge and skills but also on aligning with patient needs and live experiences. To meet these needs, user input is required in designing patient portals in specific populations dealing with distinct health care needs [81]. For example, our analysis indicated that the percentage of nonusers climbed as the ages rose: 41.1% for those aged 55 to 64 years, 49.6% for those aged 65 to 74 years, and 71.7% for those aged 70 years and older. Therefore, more studies should involve adults over 65 years to determine their patient portal design needs to increase usability. Contrary to the conventional belief that adults 65 years and older may not want to use patient portals, this age group may vary in their use based on their age cohort. It is essential in cancer care, where the burden of cancer is higher in older adults. Health care researchers focused on patient portal design and implementation will need to use community-engaged research strategies to conduct studies that will include the users and find out from them directly what will make portals helpful and attractive for them. Efforts will need to be directed toward minimizing biases in the recruitment of such studies based on age, gender, race/ethnicity, socioeconomic status, and education. Multiple studies may be needed to truly understand the needs of communities and disease populations where portals are intended to be available for users [56]. Developers of patient portals can also use some approaches used by health apps that offer user-centric interface design [82].

### Limitations

Limitations of our study include the use of self-reported data and the cross-sectional design. There is the possibility of recall bias in the frequency and use of patient portals, and the design precludes causal inference. Specifically, we cannot infer whether increased portal use causes increased PCC and vice versa [35]. We elected to examine portal use as an independent variable because of the population under consideration and other evidence suggesting the contributing role of patient accessible online records on PCC [19]. Our adjustment for confounders was limited to variables available in the HINTS data set. It is possible that unmeasured confounding affected our results. We also could not assess the type of cancer the individuals had or for what purposes individuals were accessing portals in this analysis due to small cell sizes. For example, scheduling an appointment is much different than checking for labs or communicating with a provider. It will also be challenging to address through patient portals any emotional concerns of the patient that require face-to-face direct communication. The wording of the health information self-efficacy survey item differed slightly between HINTS cycles. Based on similar distributions across cycles and our selection of the sample with only patients diagnosed with cancer, the 2 similarly worded variables were merged into a single variable. However, the 2 items may measure different dimensions of medical health information self-efficacy.

Concerning the generalizability of this study, HINTS weights only reflect certain demographic characteristics of the US population and do not take into consideration other factors that may influence individuals electing to participate in the study, which hypothetically could include factors such as greater motivation related to health and health-related constructs. The study sample includes a mix of patients with recent (<15% diagnosed less than a year ago) and distant (approximately 50% diagnosed ≥11 years ago) cancer diagnoses. Hence our results are not generalizable to more recently diagnosed patients. We also combined non-Hispanic Asians/others as our numbers in each category were too low to keep separate. Hence we could not point toward any differences based on race or ethnicity. Likewise, we were unable to compare our sample to a similar national sample of cancer survivors with respect to sociodemographic profile as these data do not exist. Last, we used the term patient portals in this paper as it is a more widely known term and most online records can be accessed via secure patient portal sign-ins. However, online medical records and patient portals could refer to different types of systems, and we cannot ascertain to which the participants were referring.

### Conclusion

In summary, PCC is a vital part of quality cancer care. Findings from this national survey suggest that increased frequency of patient portal use is associated with higher PCC and that an individual’s health information–seeking self-efficacy partially mediates this association. While the results of this study need to be replicated in future longitudinal studies, these findings suggest that interventions to encourage the adoption and use of patient portals could incorporate strategies to improve health information self-efficacy and lead to improved PCC.

## Abbreviations

HINTS: Health Information National Trends Survey
NCI: National Cancer Institute
NIH: National Institutes of Health
PCC: patient-centered communication
RRR: relative risk ratio
